# Reduction of surgical site infection using a novel intervention (ROSSINI): study protocol for a randomised controlled trial

**DOI:** 10.1186/1745-6215-12-217

**Published:** 2011-10-04

**Authors:** Thomas D Pinkney, David C Bartlett, William Hawkins, Tony Mak, Haney Youssef, Kaori Futaba, Gareth Harrison, Adrian Gheorghe, Jennifer M Bradbury, Melanie J Calvert, George Dowswell, Laura Magill, Val Redman, Sue Wilson, David Leaper, Dion G Morton

**Affiliations:** 1 Surgical Registrar, West Midlands Deanery/West Midlands Research Collaborative, UK; 2 MRC Clinical Research Fellow, NIHR Biomedical Research Unit and Centre for Liver Research, University of Birmingham, UK; 3 Primary Care Clinical Research and Trials Unit, University of Birmingham, UK; 4 Academic Department of Surgery, University Hospitals Birmingham, UK; 5 Birmingham Clinical Trials Unit, University of Birmingham, UK; 6 Emeritus Professor, University of Newcastle upon Tyne and Visiting Chair, Imperial College London, UK

## Abstract

**Background:**

Surgical site infection (SSI) is a common complication following abdominal surgery. It is associated with considerable morbidity and mortality, and its management results in significant cost to health services within both primary and secondary care. Some surgeons believe that the use of a wound-edge protection device may reduce the incidence of SSI. Whilst there is some encouraging evidence showing that such devices may lead to a reduction in SSI, there are no controlled trials of sufficient size or quality to support their routine use.

**Methods/Design:**

750 patients will be recruited from around 20 surgical units within the United Kingdom. Patients undergoing laparotomy through any major abdominal incision for any indication, elective or emergency, are eligible. Patients under the age of 18, those undergoing a laparoscopic assisted procedure or who have undergone laparotomy within the previous 3 months, and those who are unable to give informed consent will be excluded. Patients will be randomised (1:1 ratio) to the use of a wound-edge protection device or no wound-edge protection device during surgery.

Follow up will consist of blinded clinical wound reviews at 5-7 days and 30-33 days postoperatively with a self-completed questionnaire covering the intervening period. Quality of life questionnaires will be completed prior to surgery and at the subsequent wound review points and information on resource usage will also be captured.

The primary outcome measure is SSI within 30 days of surgery. Secondary outcomes include the impact of the degree of wound contamination, patient comorbidity, and operative characteristics on the efficacy of a wound-edge protection device in reducing SSI and whether the use of a wound-edge protection device has an effect on health-related quality of life or length of hospital stay and is cost-effective.

**Discussion:**

Rossini is the first multicentre observer-blinded randomised controlled trial of sufficient size and quality to establish whether the use of a wound-edge protection device in adult patients undergoing abdominal surgery leads to a lower rate of SSI. The results of this study will be used to inform current surgical practice and may potentially benefit patients undergoing surgery in the future.

**Trial registration number:**

Current Controlled Trials ISRCTN: ISRCTN40402832

## Background

Surgical site infection (SSI) is one of the most common postoperative complications and occurs in at least five percent of all patients undergoing surgery [1]. The rate of SSI is significantly higher after open abdominal surgery which can carry an SSI risk of up to 40% depending on the level of contamination [2].

SSI is associated with considerable morbidity and it has been reported that over one-third of postoperative deaths are related, at least in part, to SSI [3]. It must be appreciated, however, that the diagnosis covers a wide spectrum of clinical conditions ranging from a relatively trivial wound discharge with no other complications to a life-threatening condition. Other clinical outcomes of SSI include poor scars that are cosmetically unacceptable, such as those that are hypertrophic or keloid, persistent pain and itching and a significant impact on emotional wellbeing [4].

SSI can double the length of time a patient stays in hospital and thereby increase the costs of health care. Additional costs attributable to SSI of between £814 and £6626 per case have been reported depending on the type of surgery and the severity of the infection [5,6]. The main additional costs are related to re-operation, extra nursing care and interventions, and drug treatment costs. The indirect costs, due to loss of productivity, patient dissatisfaction and litigation, and reduced quality of life, have been studied less extensively.

Recent national guidelines concerning the prevention and treatment of SSI have been issued by the National Institute for Health and Clinical excellence (NICE) [7]. These recommendations are based on systematic reviews of best available evidence, or when minimal evidence is available the guideline development group's opinion of what constitutes good practice. Intra-operative guidance includes the role of hand decontamination, sterile gowns and drapes and antiseptic skin preparation. Wound-edge protection devices are not discussed in these guidelines, presumably due to the paucity of published research into their use and efficacy in the prevention of infection

It is notable that some adhesive plastic 'incise' drapes, favoured by some surgeons, are not recommended in the guidelines. A recent Cochrane review and meta-analysis found that they may increase the chance of an SSI [8]. Iodine-impregnated incise drapes fare better (in terms of no added risk) but still do not offer a significant improvement in wound infection rate compared with no drape at all.

### Wound-edge protection devices

Some surgeons advocate the use of a wound edge protector or 'wound guard' to reduce SSI. There are several different devices on the market but they all share the same basic design - a semi-rigid plastic ring placed into the abdomen via the laparotomy wound to which an impervious drape is circumferentially attached. This plastic drape comes up and out of the wound onto the skin surface, thus protecting the cut wound edges. The proposed mechanism of action of the device is two-fold. Firstly, they create a physical barrier between the abdominal wound edges and viscera, visceral contents, contaminated instruments and gloves - thus reducing accumulation of endogenous and exogenous bacteria on the wound edges. They also potentially reduce tissue necrosis from long procedure exposure as well as performing a degree of mechanical retraction which in turn may reduce the need for handheld mechanical retraction and the tissue damage associated therein. Smaller versions of wound-edge protection devices are also currently often used in laparoscopic-assisted resections of colorectal malignancies to prevent seeding of tumour cells into wound edges.

As mentioned above, these devices were not considered at all in the NICE guidelines and there has been minimal formal research evaluating their value. Two recent descriptive studies of their use failed to provide strong conclusions [9,10]. More encouragingly, two separate small randomised controlled trials (RCTs) comparing use of the device against standard treatment did show encouraging results [11,12], with a variable rate of reduction of up to 84% depending on degree of wound contamination. However, the trials were both single-centre and not without some shortcomings in design, in particular relating to randomisation strategy, blinding of researchers and follow-up protocols. All other research into the devices is over 25 years old and assesses older-generation devices in a different era to that of today in terms of the background advances in infection prophylaxis. Table 1 summarises the published data on the usage of wound-edge protectors [9-16]. There is no systematic review or meta-analysis of the use of a wound-edge protector. This probably accounts for the limited uptake of the device amongst the general surgeons of today. The highest level of evidence regarding the devices probably comes from a review of the Sookhai RCT [11] in the Evidence-Based Medicine journal soon after its publication [17]; the conclusion of this review was that whilst there was evidence that the device reduced SSI (particularly in operations with a higher level of contamination), wider application of the device should await the result of a more rigorously designed multicentre study.

**Table 1:** Summary of published data on wound edge protection devices

Lead Author	Year of Publication	Title	Type of Study	Comments
Horiuchi [12]	2007	Randomised, controlled investigation of the anti-infective properties of the Alexis retractor/protector of incision sites	**RCT**; 2 arms - control vs. wound-edge protector	Looks well-designed 221 patients Positive results Single centre

Kercher [9]	2004	Plastic wound protectors do not affect wound infection rates following laparoscopic-assisted colectomy	Retrospective review	Descriptive study only 141 patients Laparoscopic cases

Nakagoe [10]	2001	Minilaparotomy wound edge protector (Lap-protector): a new device	Description of technique	Descriptive study only No comparison group

Sookhai [11]	1999	Impervious wound-edge protector to reduce postoperative wound infection: a randomised, controlled trial	**RCT**; 2 arms -control vs. wound-edge protector	Looks well-designed 352 patients Positive results Single centre

Nystrom [13]	1983	A controlled trial of plastic wound ring drape to prevent contaminations and infection in colorectal surgery	**RCT**; 2 arms - control vs. wound-edge protector	140 patients No benefit found Old-generation device

Psaila [14]	1977	The role of plastic wound drapes in the prevention of wound infection following abdominal surgery	**RCT**; 3 arms - control vs. adhesive drape vs. wound-edge protector	154 patients No benefit from either device Poor follow-up

Alexander -Williams [15]	1972	Abdominal wound infections and plastic wound guards	**RCT**; 2 arms - control vs. wound-edge protector	167 patients Poor follow-up to 10 days only Inconsistent design

Maxwel [16]	1969	Abdominal wound infections and plastic drape protectors	Comparative study	No randomisation Poorly designed study 202 patients

### Issues relating to a potential randomised controlled trial of wound-edge protectors

1 No multicentre trial has been done

2 No current data explores efficacy in prevention of early versus late SSI

3 No current data explores pathogen bias in SSI prevented (or not prevented) by the device

We propose a prospective multicentre randomised controlled trial that will address all of these issues.

### Primary Hypothesis

Use of a wound-edge protection device in adults undergoing laparotomy will result in a reduced rate of surgical site infection (SSI).

## Methods/Design

### Study Design

A prospective, multicentre, observer blinded, randomised controlled trial with stratification according to baseline infection risk

### Study Setting

The study will take place in general surgical units within the NHS. Surgical units in the West Midlands are being approached as are units in London, Southampton, Plymouth and Trent. Twelve sites are being identified to collaborate in the trial initially.

### Ethical Approval

Full ethical approval has been gained from the North Staffordshire Research Ethics Committee (ref: 09/H1204/91).

### Inclusion Criteria

All adults undergoing laparotomy via midline, transverse or Kocher's incision (for any surgical indication), including both elective and emergency operations

### Exclusion Criteria

• Patients less than 18 years of age, or unable to give informed consent

• Laparoscopic-assisted cases

• Previous laparotomy within the past 3 months

### Primary outcome measure

The primary outcome is Surgical Site Infection (SSI) within 30 days of surgery.

Positive identification of SSI will be determined with use of the internationally accredited Centers for disease Control and Prevention (CDC) definitions.

We hypothesise that the use of wound-edge protection device in adults undergoing laparotomy will result in a reduced rate of surgical site infection of 50% based on evidence from Horiuchi et al, 2007 [12].

The power calculations for a range of baseline infection rates and this assumed 50% reduction in SSI rate brought about by use of the device are shown in table 2.

**Table 2:** Infection rates and sample size

Infection rate in untreated group	Expected infection rate in treated group	Overall sample size	Sample size in each arm
18%	9%	448	224

15%	7.5%	554	277

12%	6%	**710**	**355**

(80% power and 0.05 significance level)

A conservative estimate of infection rate of 12% requires a total of 710 patients, randomised 1:1 between treatment and placebo. Thus we intend to recruit 750 patients in total in order to provide adequate power should our assumptions informing the power calculation prove optimistic, and to accommodate a potential 5% dropout rate.

### Secondary Outcomes

Comparisons will be made to assess the impact of treatment relating to the following outcomes:

1) The degree of wound contamination (e.g. colorectal resections/vascular surgery)

2) Presence of major comorbidity (e.g. diabetes, obesity, smoking)

3) Health-related quality of life (QoL) to be assessed using the EQ-5D questionnaire

4) Length of stay in hospital

5) Health care utilisation and the incremental cost-effectiveness of the use of the wound-edge protection device compared to standard care

6) Adverse events

### Randomisation

The most important factor in determining risk of SSI is the type of surgery performed via the laparotomy wound and resulting degree of contamination. The standard categorisation and corresponding normal infection rates [18] are shown in table 3.

**Table 3:** Classification of potential surgical contamination

Type of Surgery	Examples	**Normal Infection Rate (%) **[18]
Clean (no viscus opened)	Adhesiolysis	1-2

Clean-contaminated (viscus opened, minimal spillage)	Right Hemicolectomy, Open Cholecystectomy	< 10

Contaminated (open viscus with spillage or inflammatory disease)	Colectomy with some spillage Resection of active Crohns disease	15-20

Dirty (pus or perforation, or incision through an abscess)	Diverticular perforation	< 40

Randomisation will therefore be stratified according to degree of contamination using a minimisation procedure following the methods proposed by Pocock and Simon (1975) [19] using a programme developed by Dr Melanie Calvert, University of Birmingham.

Please refer to the randomisation notepad (Additional file 1 Table S1) for details of operation-specific information to be collected prior to randomisation.

### Data Management

This is being supported by the Primary Care Clinical Trials Unit at the University of Birmingham. Data will be stored securely as a hard copy version and on computerised data bases (ACCESS).

### Statistical analysis

All analyses will be pre-specified and conducted according to the intention-to-treat principle with the use of SAS software (version 9.1, SAS Institute). P values other than for the primary end point are nominal.

#### Analysis of Primary and Secondary Outcomes

The primary outcome will be analysed using generalised linear models, with logit link, binomial error and with surgeon as random effects. Odds ratio 95% CI and p value will be presented. Continuous data will be analysed with the use of mixed models, which include surgeons as random effects [20]. The rates of adverse events shall be compared between groups by means of Fisher's exact test.

#### Exploratory analysis on the primary outcome

If a statistically significant result is observed for the primary outcome a parsimonious statistical model describing the predictive value of variables listed will be estimated, using a stepwise model building procedure (Additional file 2 Appendix 1). The linearity in response of continuous variables will be examined using restricted cubic splines. Interaction terms will be assessed and Akaike's Information Criterion will be used to determine the best model fit [21].

#### Cost-effectiveness Analysis

A prospective within trial cost-effectiveness analysis will be undertaken based on ITT analysis of all patients enrolled in the study. The incremental cost per quality adjusted life year (QALY) gained and incremental cost per life year gained of the alternate treatment options will be assessed during the trial to inform clinicians and policy makers of the cost effectiveness of the alternative therapies.

### Data Safety Monitoring

A steering committee developed the protocol and will provide academic leadership for the overall conduct of the trial. An independent data and safety monitoring board will be appointed who will undertake 4 interim analyses on safety and efficacy. Although the exact details of the statistical plan will be determined by the committee, it is anticipated that a truncated modified O'Brien Flemming alpha spending plan will be used for efficacy, and a non symmetrical power function will be used for safety (increasing the likelihood of early stopping for safety).

### Trial Management Protocols

Two named principle investigators (PI) will be recruited at each study site. Together they shall provide blinded wound reviews for the other PI's patients by being unaware of which arm of the study these patients belong to. The two wound reviews will occur firstly prior to discharge at day 5 to 7 post-operation and then a formal outpatient appointment or ward attendance for wound review will be arranged at or just after 30 days (day 30-33 post-operation). At this visit patients will also be asked to complete a retrospective questionnaire regarding their wound and its healing over the intervening period. This will ensure that full information is gathered about any wound infections, together with resulting treatment or hospital admissions, occurring between the day 5-7 and day 30-33 reviews. Enrolled patients will also be given contact details for the local primary investigator should any concerns by raised by the patient. Wound assessment at both stages will utilise a standardised proforma. These wound assessment tools will be pre-validated for reproducibility and ease-of-use in a pilot study to be ran prior to full trial launch.

QOL data will be assessed using the EQ-5D pre-operatively, at 5-7 days, at the 30-33 day follow-up visit.

We also envisage the involvement of clinical nurse specialists at most sites. Their clinic appointments are generally more accessible and it may be feasible to utilise these for the second formal (outpatient) wound review. Clinical nurse specialists are generally experienced in assessing post-operative wounds themselves and they may also therefore provide a second-line cohort of blinded wound reviewers if the relevant PI is not available.

### Study Process

**Figure 1:**
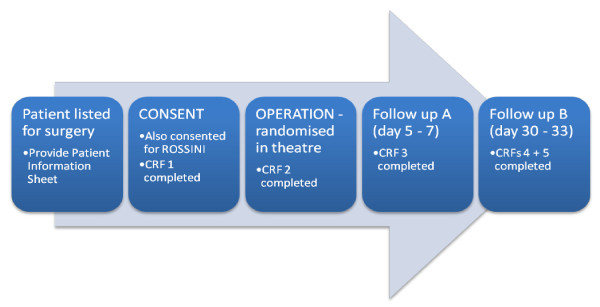
**Flowchart Illustrating Study Process**.

Data will be recorded on a number of case report forms which are detailed in Additional file 3 Appendix 2. Figure 1 gives an overview of the study process.

1. Informed consent for participation in the study is obtained preoperatively, ideally at the same setting where (normal) consent for the intended surgical procedure is obtained > 24 hrs prior to surgery (except in an emergency setting). Patients will ideally have been previously given a patient information sheet at the time of listing for their operation.

2. After induction of anaesthesia at the time of surgery the patient is allocated to either the wound-edge protector group or the control (no protector) group by the surgeon, using a secure 24 hour internet-based randomisation service - provided by the UKCRC Accredited Primary Care Clinical Trials Unit, University of Birmingham.

3. Normal local policy for systemic antibiotic prophylaxis is followed.

4. Standard routine skin preparation is undertaken according to local policy.

5. Antiseptic-soaked towels on wound edges may also be used in either group.

6. The operation is carried out as normal, with removal of the wound guard (if applicable) as late as possible prior to wound closure.

## List of Abbreviations

SSI: Surgical Site Infection; NICE: National Institute for Health and Clinical Excellence; RCT: Randomised Controlled Trial; NHS: National Health Service; CDC: Centers for Disease Control and Prevention; QoL: Quality of Life; QALY: Quality Adjusted Life Year; PI: Principle Investigator; UKCRC: United Kingdom Clinical Research Collaboration; CRF: Case Report Form; BMI: Body Mass Index.

## Competing interests

The authors declare that they have no competing interests.

## Authors' contributions

TP is the chief investigator and has led and been involved in all stages of the study design and together with DB, WH, TM, HY, KF and GH participated in the writing of the protocol, submission to the funding body and application to ethics as well as identifying and approaching sites for recruitment. AG assisted in writing the protocol, in particular the cost effectiveness part of the study, and has developed pathways for measuring patient costs during the study. JB assisted participating sites with local ethical/R&D approval, coordinated site opening and participated in the production of the case report forms. MC, GD, SW provided guidance on trial design and statistical analysis, drafted sections of the protocol and were co-investigators in the submission to the funding body. LM oversaw the ethics application and assisted in drafting the protocol and case report forms. VR is the trial manager and participated in the design of the case report forms, site opening and applying for ethical approval. DL provided guidance on trial design and participated in the writing of the protocol. DM has provided senior support throughout the design of the study and writing the protocol. All authors read and approved the final manuscript.

## Supplementary Material

Additional file 1 **Table S1**. Randomisation Notepad. Click here for file

Additional file 2 **Appendix 1**. Exploratory analysis on the primary outcome. Click here for file

Additional file 3 **Appendix 2**. Case report form definitions. Click here for file
