# Supplementary material for: Reduction of surgical site infection using a novel intervention (ROSSINI): study protocol for a randomised controlled trial
Source: Trials. 2011 Oct 4;12:217. doi: 10.1186/1745-6215-12-217 (PMC3201898; doi:10.1186/1745-6215-12-217)
Supplement: Additional file 1 — Table S1. Randomisation Notepad. [file 1745-6215-12-217-S1.DOC]

**Randomisation Notepad**

*Note – the randomisation will take place online – these questions will be asked ‘electronically’ with a series of tick-boxes before the group (CONTROL or INTERVENTION) is allocated automatically*

| **Date of Randomisation** |  | d | d | m | m | y | y | y | y |
| --- | --- | --- | --- | --- | --- | --- | --- | --- | --- |

**Part A – Identifying Details**

| **ROSSINI** trial number |  |  |  |  |  | **Centre** number |  |  |  |  | Randomising clinician |
| --- | --- | --- | --- | --- | --- | --- | --- | --- | --- | --- | --- |
|  | n | n | n | n |  |  | n | n | n | n |  |

|  | | | | | | | | Date of birth: | | | | | d | d | m | m | y | y | y | y |  |
| --- | --- | --- | --- | --- | --- | --- | --- | --- | --- | --- | --- | --- | --- | --- | --- | --- | --- | --- | --- | --- | --- |
|  | | | | | | | |  | | | | | | | | | | | | | |
| NHS number: |  |  |  |  |  |  |  | |  |  |  |  | | | | | | | | | |
|  | n | n | n | n | n | n | n | | n | n | n |  | | | | | | | | | |

**Part B – Eligibility Criteria**

| 1 | Do you plan to open any viscus? (Including any part of the biliary, alimentary or genitourinary tracts? | | | | | | | | | | | | | | | | | | | | | | | | | | | | |  | |
| --- | --- | --- | --- | --- | --- | --- | --- | --- | --- | --- | --- | --- | --- | --- | --- | --- | --- | --- | --- | --- | --- | --- | --- | --- | --- | --- | --- | --- | --- | --- | --- |
|  |  | | | | | | | | | | | | | | | | | | | | | | | | | | | | |  | |
|  | Yes |  |  | | No | | |  | | Not sure | | | | | |  | | | |  | | | | | | | | | |  | |
|  | *(Note for protocol – ‘NOT SURE’ counts as ‘NO’ for the purposes of stratification of randomisation)* | | | | | | | | | | | | | | | | | | | | | | | | | | | | |  | |
|  |  | | | | | | | | | | | | | | | | | | | | | | | | | | | | |  | |
| 2 | Do you plan on creating a stoma? | | | | | | | | | | | | | | | | | | | | | | | | | | | | |  | |
|  | Yes |  |  | | No | | |  | | Not sure | | | | | |  | | | |  | | | | | | | | | |  | |
|  | *(Note for protocol – ‘NOT SURE’ counts as ‘NO’ for the purposes of stratification of randomisation)* | | | | | | | | | | | | | | | | | | | | | | | | | | | | |  | |
|  |  | | | | | | | | | | | | | | | | | | | | | | | | | | | | |  | |
| 3 | Please identify the NCEPOD classification of this operation: | | | | | | | | | | | | | | | | | | | | | | | | | | | | |  | |
|  |  | | | | | | | | | | | | | | | | | | | | | | | | | | | | |  | |
|  | Immediate (life saving) |  |  | | | Urgent | | | | | | | | | |  | | | |  | | | | | | | | | |  | |
|  |  |  |  | | | | | | | | | | | | | | | | | | | | | | | | | | |  | |
|  | Expedited |  |  | | | Elective | | | | | | | | | |  | | | |  | | | | | | | | | |  | |
|  | *(Note for protocol – first 3 categories grouped together as ‘non-elective’ for purposes of stratification of randomisation)* | | | | | | | | | | | | | | | | | | | | | | | | | | | | |  | |
| 4 | Is this a laparoscopic-assisted case? | | | | | | | | | | | | | | | | | | | | | | | | | | | | |  | |
|  | Yes |  |  | No | | | |  | |  | | | | | *If shaded boxes are ticked, patient* ***not eligible*** *for ROSSINI* | | | | | | | | | | | | | | |  | |
| 5 | Has the patient given informed consent | | | | | | | | | | | | | |  | |
|  | Yes |  |  | No | | | |  | |  | | | | |  | |
|  |  | | | | | | | |  | | | |  | |  | | | |  | | |  | |  | |  |  |  |  |  |  |
| 6 | Which version of the consent form was used? | | | | | | | | Version: | | v | ● | | v | d | | d | m | | | m | | y | | y | | y | y |  | | |
|  |  | | | | | | | | | | | | | |  | | | | | | | | | | | | | | |  | |
| 7 | What is the name of the clinician taking consent? | | | | | |  | | | | | | | | | | | | | | | | | | | | | | |  | |

**Part C – Randomisation – Treatment Allocation**

| Control (No wound guard) |  |  |
| --- | --- | --- |
|  |  |  |
| Intervention (Wound guard) |  |  |
