# Supplementary material for: Reduction of surgical site infection using a novel intervention (ROSSINI): study protocol for a randomised controlled trial
Source: Trials. 2011 Oct 4;12:217. doi: 10.1186/1745-6215-12-217 (PMC3201898; doi:10.1186/1745-6215-12-217)
Supplement: Additional file 2 — Appendix 1. Exploratory analysis on the primary outcome. [file 1745-6215-12-217-S2.DOC]

**Appendix 1: Exploratory analysis on Primary Outcome**

The following variables are associated with infection rate and will be assessed using a prognostic model:

- Degree of wound contamination (table 3)
- Diabetes
- Immunocompromised status including steroid medications
- Presence of Cancer
- Age
- Obesity
- Low BMI/cachexia
- Smoking
- ASA grade (which may indirectly estimate other comorbidities)
- Length of surgical procedure
- Jaundice
- Previous positive MRSA culture (at any time historically)
- Interruptions to patient’s homeostasis during surgery (e.g. period of hypoxia or hypotension) lasting for at least 10 minutes
- Which prophylactic antibiotics given
- Post-op antibiotics
- Grade of surgeon closing wound
