# Supplementary material for: Reduction of surgical site infection using a novel intervention (ROSSINI): study protocol for a randomised controlled trial
Source: Trials. 2011 Oct 4;12:217. doi: 10.1186/1745-6215-12-217 (PMC3201898; doi:10.1186/1745-6215-12-217)
Supplement: Additional file 3 — Appendix 2. Case report form definitions. [file 1745-6215-12-217-S3.DOC]

**Appendix 2: Case Report Form (CRF) definitions:**

**Case Report Form 1** Basic demographic data including age, sex, BMI, comorbidity etc

**Case Report Form 2** Theatre data including study arm, operative details etc

**Case Report Form 3**  Blinded first wound review

**Case Report Form 4** Patient-completed retrospective questionnaire

**Case Report Form 5** Blinded second review

Also to be completed but not shown on study flowchart:

**Case Report Form 6**  Resource usage – completed after wound review visits are complete

**EuroQOL (EQ-5D)** questionnaire to be completed at a) consent stage

b) 5-7 days (whilst inpatient)

c) 30-33 days (at 2nd wound review)
